# Phylogenetic position of a whale-fall lancelet (Cephalochordata) inferred from whole mitochondrial genome sequences

**DOI:** 10.1186/1471-2148-7-127

**Published:** 2007-07-31

**Authors:** Takeshi Kon, Masahiro Nohara, Yusuke Yamanoue, Yoshihiro Fujiwara, Mutsumi Nishida, Teruaki Nishikawa

**Affiliations:** 1 Department of Marine Bioscience, Ocean Research Institute, the University of Tokyo, 1-15-1 Minamidai, Nakano, Tokyo 164-8639, Japan; 2 Yokohama R&D Center, HITEC Co., Ltd., 2-20-5 Minamisaiwai, Nishi, Yokohama, Kanagawa 220-0005, Japan; 3 Graduate School of Agricultural and Life Sciences, the University of Tokyo, 1-1-1 Yayoi, Bunkyo, Tokyo 113-8657, Japan; 4 Extremobiosphere Research Center, Japan Agency for Marine-Earth Science and Technology (JAMSTEC), 2-15 Natsushima-cho, Yokosuka, Kanagawa 237-0061, Japan; 5 The Nagoya University Museum, Nagoya University, Chikusa Aichi 464-8601, Japan

## Abstract

**Background:**

The lancelet *Asymmetron inferum *(subphylum Cephalochordata) was recently discovered on the ocean floor off the southwest coast of Japan at a depth of 229 m, in an anaerobic and sulfide-rich environment caused by decomposing bodies of the sperm whale *Physeter macrocephalus*. This deep sulfide-rich habitat of *A. inferum *is unique among the lancelets. The distinguishing adaptation of this species to such an extraordinary habitat can be considered in a phylogenetic framework. As the first step of reconstruction of the evolutionary processes in this species, we investigated its phylogenetic position based on 11 whole mitochondrial genome sequences including the newly determined ones of the whale-fall lancelet *A. inferum *and two coral-reef congeners.

**Results:**

Our phylogenetic analyses showed that extant lancelets are clustered into two major clades, the *Asymmetron *clade and the *Epigonichthys *+ *Branchiostoma *clade. *A. inferum *was in the former and placed in the sister group to *A. lucayanum *complex. The divergence time between *A. inferum *and *A. lucayanum *complex was estimated to be 115 Mya using the penalized likelihood (PL) method or 97 Mya using the nonparametric rate smoothing (NPRS) method (the middle Cretaceous). These are far older than the first appearance of large whales (the middle Eocene, 40 Mya). We also discovered that *A. inferum *mitogenome (mitochondrial genome) has been subjected to large-scale gene rearrangements, one feature of rearrangements being unique among the lancelets and two features shared with *A. lucayanum *complex.

**Conclusion:**

Our study supports the monophyly of genus *Asymmetron *assumed on the basis of the morphological characters. Furthermore, the features of the *A. inferum *mitogenome expand our knowledge of variation within cephalochordate mitogenomes, adding a new case of transposition and inversion of the *trnQ *gene. Our divergence time estimation suggests that *A. inferum *remained a member of the Mesozoic and the early Cenozoic large vertebrate-fall communities before shifting to become a whale-fall specialist.

## Background

The subphylum Cephalochordata (lancelets), one of the basal groups of living chordates [1,2], appears to have maintained its basic body plan for several hundred million years [3,4]. Lancelets are widely distributed in tropical and temperate shallow seas and consist of three genera (*Branchiostoma*, *Epigonichthys*, and *Asymmetron*) with more than 30 known living species [5-8]. Adults are benthic, inhabiting sandy and shell/sand bottoms of clear seawater, while larvae are planktonic in both inshore and offshore areas (ca. 1.5 to 4 months duration) [5,9-11]. For a long time, it has been believed that the lancelets prefer such aerobic conditions, whereas recently a new lancelet *Asymmetron inferum *was discovered in an anaerobic and sulfide-rich bottom [7]. This species is only distinguishable morphologically from congeners by the number of myomeres [7].

**Figure 1:**
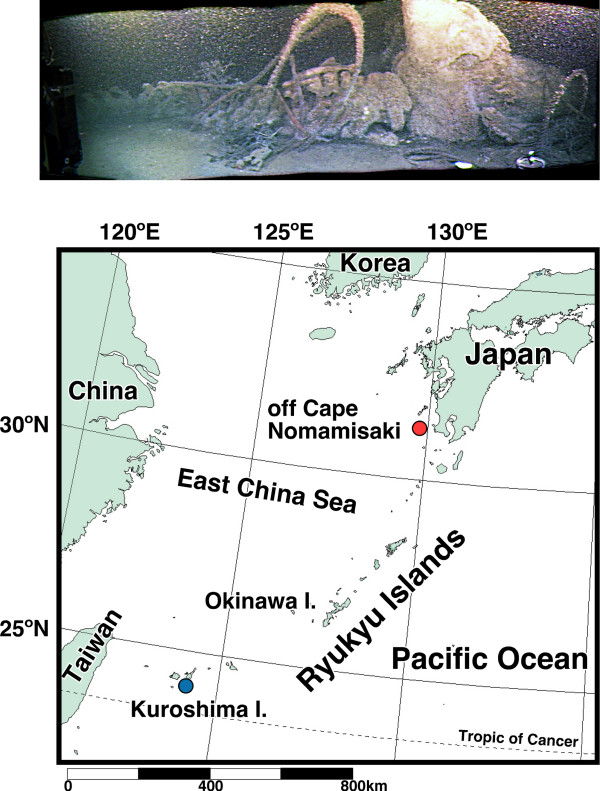
**Submerged whale carcass (upper) and map of sampling sites of *Asymmetron *in Japan (lower)**. (Upper) Video still of skeletonized sperm whale carcass at a depth of 229 m in 2003. (Lower) Localities are color-coded: red, sampling site of *A. inferum*; blue, *Asymmetron *sp. A. The third examined species *Asymmetron *sp. C was collected in Bermuda, Atlantic Ocean (see [8], site not shown).

In July 2003, during the Hyper-Dolphin/Natsushima cruise of the Japan Marine Science and Technology Center (JAMSTEC), 26 specimens of *A. inferum *were collected from bottom sand subjacent to the decomposing bones of the sperm whale *Physeter macrocephalus *at a depth of 229 m, off Cape Nomamisaki, Kyushu Island, southwest Japan (Fig. 1). Twelve dead whales that had been stranded on the southwestern coast of Kyushu Island were submerged in February 2002, and then were observed to study the faunal composition and succession of the biological communities associated with whale-falls [12]. The submerged bodies of whales on the floor provided rich pickings that resulted in substantial amounts of organic material, lipids, and sulfides for dense biological community comprising of the mytillid mussels *Adipicola pacifica *and *A. crypta *[13,14] and the bone-eating marine worm *Osedax japonicus *[15]. *A. inferum *is also a member of this community [12].

This deep (>200 m) and sulfide-rich habitat is unique to *A. inferum *in the lancelets. A sulfide-rich environment – including hydrogen sulfide – is generally toxic to macro-organisms. Therefore, the adaptation of this species to such a lethal environment is an intriguing phenomenon, but the phylogenetic framework for understanding its processes has not yet been established. Nohara et al. [16] conducted molecular phylogenetic analysis of the intra-relationships of some lancelets using whole mitochondrial genome (mitogenome) sequences, but *A. inferum *and two congeneric species of *A. lucayanum *complex [8] were not included. To know the phylogenetic position of the whale-fall lancelet *A. inferum*, we determined the whole mitogenome sequences from three species of the genus *Asymmetron *including this species (*A. inferum*, *Asymmetron *sp. A, and *Asymmetron *sp. C [=*A. lucayanum sensu stricto*] in *A. lucayanum *complex [8]) to compare with the published data for Nohara's *A. lucayanum *[16] (called here as *Asymmetron *sp. B in *A. lucayanum *complex [8]), *Epigonichthys maldivensis*, *Branchiostoma belcheri*, *B. lanceolatum *and *B. floridae *as well as three other deuterostomes as outgroups.

## Results

### Features of *A. inferum *mitogenome

**Figure 2:**
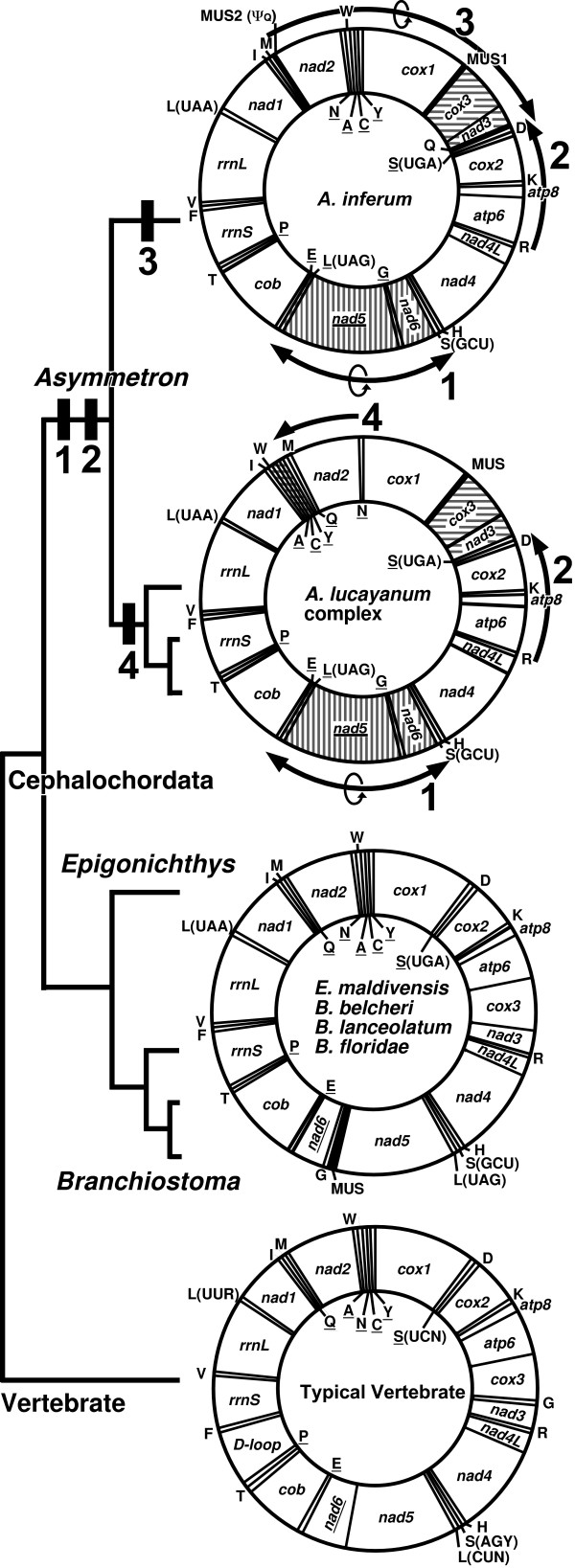
**Gene rearrangements found in the lancelets**. Gene order rearrangement events of lancelet mitogenomes were mapped onto the phylogenetic tree. Bars (1–4) correspond to the unique gene order rearrangements as shown in right genome maps. Names of 13 mitochondrial protein genes, abbreviated as in text. Twenty-two tRNA genes, denoted by standard single letter amino acid code. MUS in the maps refers to major unassignable sequence in the mitogenome. Genes encoded on light strand of the mitogenome underlined.

The nucleotide sequences from the mitogenomes of the three *Asymmetron *species have been deposited in DDBJ/EMBL/GenBank under the accession numbers of AP009352 for *A. inferum*, AP009353 for *Asymmetron *sp. A, and AP009354 for *Asymmetron *sp. C (=*A. lucayanum sensu stricto*). The total lengths of the *A. inferum*, *Asymmetron *sp. A, and *Asymmetron *sp. C mitogenomes were 15,084, 15,050, and 15,100 bp, respectively. Mitogenomes of the mentioned species of *Asymmetron*, each contained 37 genes for large- and small-subunit ribosomal RNAs (*rrnL *and *rrnS*, respectively), 22 transfer RNAs (*trnX*; X is the standard single-letter amino acid code), and 13 proteins (ATP synthetase subunits 6 and 8 [*atp6 *and *atp8*], cytochrome *c *oxidase subunits I-III [*cox1-3*], NADH dehydrogenase subunits 1–6 and 4L [*nad1-6 and 4L*], and cytochrome *b *apoenzyme [*cob*]), as is generally the case with metazoan mitogenomes (Fig. 2, Tables 1 and 2). The mitogenome organization of *Asymmetron *sp. A and *Asymmetron *sp. C was identical to that of *Asymmetron *sp. B (= Nohara's *A. lucayanum *[16]), while that of *A. inferum *was unique among the lancelets.

**Table 1:** Location of features in the mitogenome of *Asymmetron inferum*.

Features	Position number		Size (bp)	Codon			Intergenic nucleotides
						
	From	To		Start	Stop	anti-codon	
*cox1*	1	1548	1548	GTG	TAA		45
*cox3*	1594	2381	788	ATG	TA-		0
*nad3*	2382	2735	354	ATG	TAA		8
*trnQ*	2744	2812	69			TTG	3
*trnS*(UGA)	2816	2886	71			TGA	14
*trnD*	2901	2967	67			GTC	0
*cox2*	2968	3658	691	ATG	T--		0
*trnK*	3659	3722	64			TTT	5
*atp8*	3723	3896	174	GTG	TAA		-7
*atp6*	3890	4574	685	ATG	T--		0
*trnR*	4575	4638	64			TCG	0
*nad4L*	4639	4913	275	ATG	TA-		0
*nad4*	4914	6272	1359	ATG	TAA		9
*trnH*	6282	6346	65			GTG	0
*trnS*(GCU)	6347	6412	66			GCT	3
*nad6*	6416	6916	501	ATG	TAA		-15
*trnG*	6902	6967	66			TCC	0
*nad5*	6968	8759	1792	GTG	T--		0
*trnL*(UAG)	8760	8826	67			TAG	5
*trnE*	8832	8895	64			TTC	1
*cob*	8897	10039	1143	ATG	TAA		0
*trnT*	10040	10104	65			TGT	-1
*trnP*	10104	10168	65			TGG	0
*rrnS*	10169	11022	854				0
*trnF*	11023	11087	65			GAA	0
*trnV*	11088	11154	67			TAC	0
*rrnL*	11155	12514	1360				0
*trnL*(UAA)	12515	12584	70			TAA	0
*nad1*	12585	13527	943	ATG	T--		0
*trnI*	13528	13593	66			GAT	1
*trnM*	13595	13661	67			CAT	48
*nad2*	13710	14750	1041	ATG	TAA		-8
*trnN*	14743	14808	66			GTT	1
*trnW*	14810	14877	68			TCA	3
*trnA*	14881	14943	63			TGC	5
*trnC*	14949	15005	57			GCA	0
*trnY*	15006	15072	67			GTA	12

Genes encoded on light strand of the mitogenome underlined.

**Table 2:** Location of features in the mitogenome of two species of *Asymmetron lucayanum *complex.

	*A. lucayanum *complex sp. A							*A. lucayanum *complex sp. C						
		
	Position number		Size (bp)	Codon		anti-codon	Intergenic nucleotides	Position number		Size (bp)	Codon		anti-codon	Intergenic nucleotides
										
Features	From	To		start	stop			From	To		start	stop		
*cox1*	1	1548	1548	GTG	TAA		34	1	1548	1548	GTG	TAA		43
*cox3*	1583	2371	789	ATG	TAG		15	1592	2380	789	ATG	TAA		13
*nad3*	2387	2740	354	ATG	TAG		8	2394	2747	354	ATG	TAG		11
*trnS*(UGA)	2749	2819	71			TGA	11	2759	2829	71			TGA	14
*trnD*	2831	2898	68			GTC	0	2844	2912	69			GTC	0
*cox2*	2899	3589	691	ATG	T--		0	2913	3603	691	ATG	T--		0
*trnK*	3590	3652	63			TTT	0	3604	3667	64			TTT	0
*atp8*	3653	3826	174	GTG	TAA		-7	3668	3841	174	GTG	TAA		-7
*atp6*	3820	4504	685	ATG	T--		0	3835	4519	685	ATG	T--		0
*trnR*	4505	4568	64			TCG	0	4520	4583	64			TCG	0
*nad4L*	4569	4843	275	ATG	TA-		0	4584	4858	275	ATG	TA-		0
*nad4*	4844	6202	1359	ATG	TAA		1	4859	6217	1359	ATG	TAA		1
*trnH*	6204	6269	66			GTG	0	6219	6284	66			GTG	0
*trnS*(GCU)	6270	6335	66			GCT	1	6285	6350	66			GCT	1
*nad6*	6337	6840	504	ATG	TAA		-15	6352	6855	504	ATG	TAA		-15
*trnG*	6826	6892	67			TCC	0	6841	6906	66			TCC	0
*nad5*	6893	8681	1789	ATG	T--		0	6907	8695	1789	GTG	T--		0
*trnL*(UAG)	8682	8749	68			TAG	6	8696	8762	67			TAG	9
*trnE*	8756	8819	64			TTC	5	8772	8836	65			TTC	4
*cob*	8825	9967	1143	ATG	TAA		0	8841	9983	1143	ATG	TAG		0
*trnT*	9968	10037	70			TGT	0	9984	10053	70			TGT	0
*trnP*	10038	10101	64			TGG	0	10054	10116	63			TGG	0
*rrnS*	10102	10943	842				0	10117	10964	848				0
*trnF*	10944	11006	63			GAA	0	10965	11027	63			GAA	0
*trnV*	11007	11073	67			TAC	0	11028	11094	67			TAC	0
*rrnL*	11074	12437	1364				0	11095	12460	1366				0
*trnL*(UAA)	12438	12506	69			TAA	0	12461	12530	70			TAA	0
*nad1*	12507	13449	943	GTG	T--		0	12531	13473	943	ATG	T--		1
*trnI*	13450	13515	66			GAT	15	13475	13540	66			GAT	20
*trnW*	13531	13598	68			TCA	9	13561	13629	69			TCA	3
*trnA*	13608	13670	63			TGC	3	13633	13695	63			TGC	2
*trnC*	13674	13727	54			GCA	0	13698	13752	55			GCA	0
*trnY*	13728	13792	65			GTA	12	13753	13818	66			GTA	42
*trnM*	13805	13871	67			CAT	-1	13861	13927	67			CAT	-1
*trnQ*	13871	13939	69			TTG	2	13927	13995	69			TTG	1
*nad2*	13942	14982	1041	ATG	TAA		-8	13997	15037	1041	ATG	TAA		-8
*trnN*	14975	15041	67			GTT	9	15030	15095	66			GTT	5

Genes encoded on light strand of the mitogenome underlined.

The mitogenome of *A. inferum *contained 13 protein-coding genes, of which one reading-frame overlapped on the same strand (*atp8 *and *atp6 *shared 7 nucleotides). Ten protein-coding genes (*atp6*, *cob*, *cox2*, *cox3*, *nad1-4*, *nad4L*, and *nad6*) started with ATG, the other protein genes (*atp8*, *cox1*, and *nad5*) with GTG (Table 1). Stop codons of protein-coding genes were TAA in the *atp8*, *cob*, *cox1*, *nad2-4*, and *nad6*; TA in the *cox3*, and *nad4L*; and T in the *atp6*, *cox2*, *nad1*, and *nad5*. For those genes with an incomplete stop codon, the transcripts would be modified to form the complete termination signal UAA by polyadenylation after cleavage of the polycistronic RNA, as demonstrated for other metazoan mitogenomes [17]. There were 22 tRNA genes, which were clustered or individually scattered in the genome. The tRNA genes ranged in size from 57 to 71 nucleotides, large enough for the encoded tRNAs to fold into the cloverleaf secondary structure characteristic of tRNAs. The *trnS*(*gcu*) and *trnC *lacked the DHU arm, like *Branchiostoma floridae *[18]. The ribosomal genes (*rrnS *and *rrnL*) of *A. inferum *were 854 bp and 1360 bp, respectively. They were located, as in other lancelets, between the *trnP *and *trnL*(*uaa*) genes, being separated by the *trnF *and *trnV *genes. There were two major unassignable regions (MUS) longer than 40 bp in the *A. inferum *mitogenome. One of the regions (MUS1, 45 bp) was located at the identical position to MUS in the mitogenomes of the *A. lucayanum *complex (between the *cox1 *and *cox3 *genes) [16]. The other region (MUS2, 48 bp), located between the *trnM *and *nad2 *genes, was unique among the lancelet mitogenomes.

### Phylogenetic relationships

**Figure 3:**
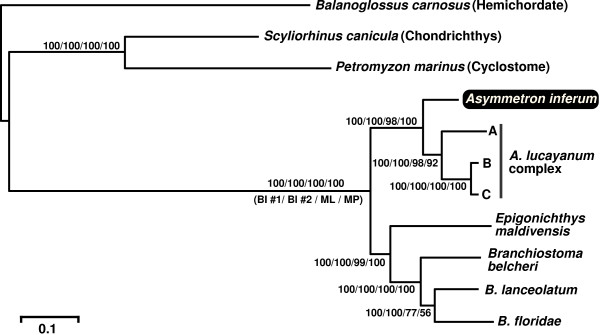
**Phylogenetic relationships of the lancelets**. Bayesian inference (BI) tree for eight lancelets with three outgroups (one cyclostome, one chondrichthys, and one hemichordate) based on whole mitogenome sequences (dataset #2). Numbers on branches refer to BI posterior probabilities (dataset #1 and #2), ML bootstraps, and MP bootstraps, respectively.

Partitioned Bayesian inference (BI) phylogenetic analysis of the 11 mitogenomes from the concatenated nucleotide sequences from 13 protein-coding genes, 22 tRNA genes, plus 2 rRNA genes (dataset #1) under the general time reversible model with gamma correction and invariable-site assumption (GTR + *I *+ *Γ*) [19] yielded a topology with resolution of the branching pattern among lancelets. All nodes were supported by higher Bayesian posterior probabilities (100%). BI analysis using dataset #2 (triplets converted amino acid sequences) under mtREV + *I *+ *Γ *[20] (for protein-coding genes) and GTR + *I *+ *Γ *(for tRNA and rRNA genes) models produced the same tree topology (Fig. 3). All nodes were supported by higher Bayesian posterior probabilities (100%). Maximum-likelihood (ML) analysis using dataset #1 under the transversional substitution model with gamma correction and invariable-site assumption (TVM + *I *+ *Γ*) produced exactly the same tree topology as that found in the BI analyses (figure not shown). Most nodes were supported by high bootstrap probabilities (>98%), with the exception of a clade comprising *B. lanceolatum *and *B. floridae *(77%). Heuristic maximum parsimony (MP) analysis of the dataset #1 also produced the identical tree topology as in the above analyses (figure not shown). The MP analysis yielded the single most parsimonious tree, with a length of 23,178 steps (consistency index [CI], 0.605; retention index [RI], 0.455; and rescaled consistency index [RC], 0.275). Most nodes were supported by high bootstrap values (>92%), with the exception of the same clade (*B. lanceolatum *+ *B. floridae*) as in ML analysis (56%).

### Divergence time estimation

**Figure 4:**
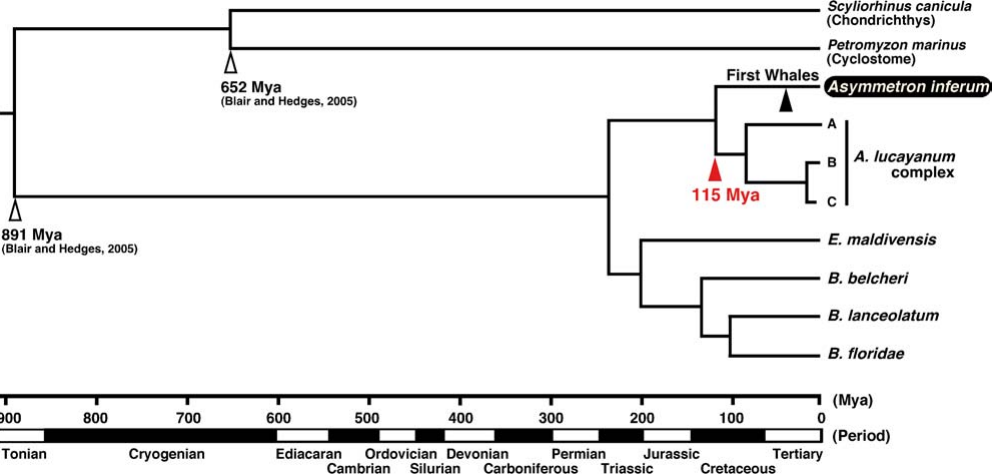
**Divergence time estimation of lancelets based on penalized likelihood (PL) method**. Time tree from semiparametric rate smoothing (penalized likelihood, PL) based on the BI tree (Fig. 3). Scale bar shows time scale resulting from calibration using the divergence time between Cephalochordata and Vertebrata (+ Urochordata) (891 Mya) and between Agnatha (Cyclostome) and Gnathostomes (Chondrichthys) (652 Mya) [1]. Arrows indicate calibration point (open head), divergence time between *A. inferum *and *A. lucayanum *complex (red solid head), and first appearance of large whales (black solid head).

**Figure 5:**
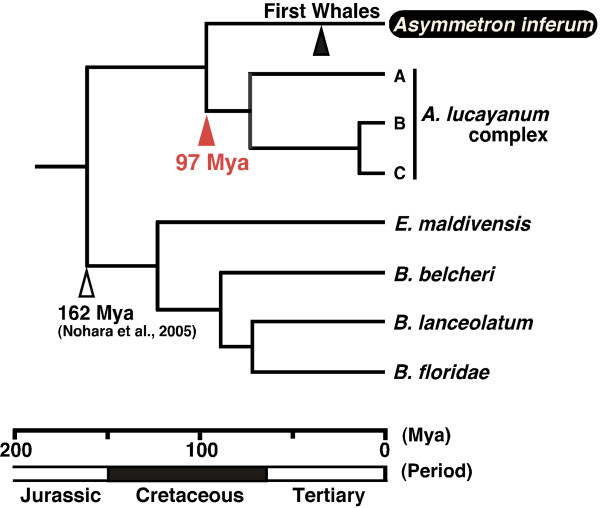
**Divergence time estimation of lancelets based on nonparametric rate smoothing (NPRS) method**. Ultrametric tree based on nonparametric rate smoothing (NPRS) analysis of the ML tree. Scale bar shows time scale resulting from calibration using the divergence time between *Asymmetron *and the other genera (162 Mya) [16]. Arrows indicate calibration points (open head), divergence time between *A. inferum *and *A. lucayanum *complex (red solid head), and first appearance of large whales (black solid head).

As a high rate of heterogeneity among lineages of lancelets was observed by the two-cluster test [21], we used two different molecular dating methods, the penalized likelihood (PL) [22]) based on the BI tree (dataset #2) and the nonparametric rate smoothing (NPRS) [23] based on the ML tree (dataset #1). The divergence time between *A. inferum *and *A. lucayanum *complex was estimated to be 115 Mya using PL method (Fig. 4) or 97 Mya using NPRS method (Fig. 5).

## Discussion

### Phylogenetic position of *A. inferum*

Our phylogenetic analyses of mitogenome sequences for eight lancelets using Bayesian inference (BI), maximum likelihood (ML), and maximum parsimony (MP) methods show that extant lancelets are clustered into two major clades: the *Asymmetron *clade and the *Epigonichthys *+ *Branchiostoma *clade (Fig. 3). This result is consistent with the findings of Nohara et al. [16]. The topology of the resulting trees implies that the asymmetrical arrangement of gonads seen paraphyletically in the genus *Asymmetron *and *Epigonichthys *can be regarded as a plesiomorphic feature, supporting the hypothesis that the gonadal symmetry in the genus *Branchiostoma *was derived from the asymmetric *Epigonichthys*-like ancestor [16]. Nishikawa [7] recovered the genus *Asymmetron *as a valid genus distinct from another valid genus *Epigonichthys *based on four morphological characters as follows: the elongated urostyloid process, marked metapleuran asymmetry, intercirral membrane with abrupt height change between lateral and ventral ones, and posterior shift of the cirral skeletal ring [24] with its anterodorsal extremity located at (or sometimes behind) the fourth myomere. The present molecular phylogeny shows that these morphological characters of the *Asymmetron *species are phylogenetically informative, supporting his claim. Although the *A. lucayanum *complex includes deep divergence (*p*-distance = 19% in *cox1 *of mitogenome [8]), *A. inferum *is placed on the sister group to this species complex, not nested within it (Fig. 3). This result indicates that the whale-fall lancelet *A. inferum *was diverged from the circumtropical lancelet *A. lucayanum *complex before the ancient separation in this complex [8].

### Gene rearrangement of *A. inferum*

We discovered that the *A. inferum *mitogenome has been subjected to large-scale gene rearrangements. To elucidate the relative timing of these gene rearrangements, we mapped gene orders of the lancelet mitogenome onto the phylogenetic tree (Fig. 2). Gene orders of *Epigonichthys *and *Branchiostoma *are similar to that of typical vertebrate mitogenomes [25], with the exception of slight differences in the location of four tRNA genes [18]. Therefore, it is reasonable to assume that the ancestral lancelet gene order is almost the same as that of the *Epigonichthys *and *Branchiostoma*. The *A. inferum *mitogenome has three novel gene positions: (1) an inversion extending from the *trnL*(*uag*) to *nad6 *genes; (2) transposition of the *cox3 *and *nad3 *genes from between the *atp6 *and the *trnR *genes to between the *cox1 *and *trnS*(*uga*) genes; and (3) an inversion of the *trnQ *gene and its transposition from between the *trnM *and *nad2 *genes to between the *nad3 *and the *trnS*(*uga*) genes. The gene order of this mitogenome can be parsimoniously explained by the following steps: events (1) and (2) occurred in the ancestor of all *Asymmetron *species, event (3) subsequently occurred in the lineage of *A. inferum*, and event (4) (see [16]) occurred in the ancestor of *A. lucayanum *complex (Fig. 2). In other words, novel gene positions (1) and (2) represent molecular synapomorphies shared among the *Asymmetron *species, and novel gene position (4) is the synapomorphy of *A. lucayanum *species complex.

**Figure 6:**
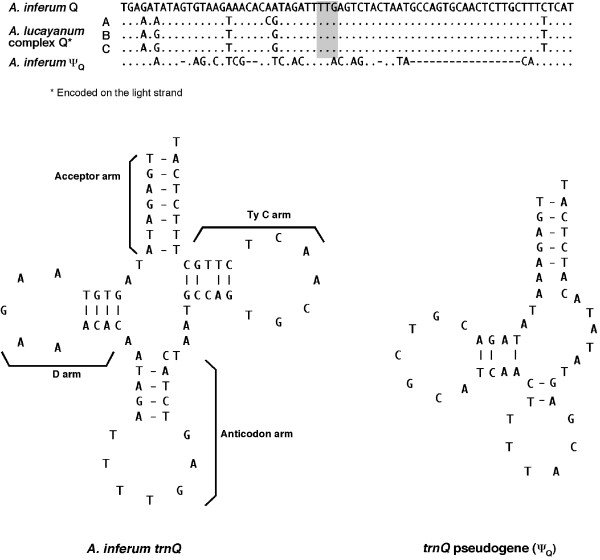
**Aligned sequences (upper) and potential secondary structures (lower) of *trnQ *gene and putative *trnQ *pseudogene**. (Upper) Aligned sequences of the four *trnQ *genes (Q) of four species *Asymmetron *and a putative *trnQ *pseudogene (ψ_Q_) from *A. inferum*. Dots indicate sequence identity with the first sequences (*trnQ* gene of *A. inferum*), and dashes indicate alignment gaps. Sequence in grey box indicates anticodon. (Lower) Potential secondary structures of *trnQ *gene and putative *trnQ *pseudogene (ψ_Q_) in *A. inferum*.

Gene position (3) and existence of MUS2 are unique to *A. inferum*. MUS2, located at the identical position of *trnQ *in the mitogenomes of *A. lucayanum *complex, appears to be a *trnQ *pseudogene because of its sequence similarity to the acceptor and anticodon arm regions of *trnQ *of *A. inferum *and complement sequences of that of *A. lucayanum *complex (Fig. 6). Therefore, gene arrangement pattern (3) may have resulted from two events as follows: the inversion of *trnQ *gene occurred at the original position by recombination of the mitogenome as a possible cause for pattern (1) [16], followed by transposition, involving a tandem genomic duplication and subsequent random deletions of the duplicated part, as invoked for many cases of gene rearrangement in animal mitogenomes ([25] and references therein).

### When and how did *A. inferum *become a whale-fall specialist?

*A. inferum *is considered as whale-fall specialist. This species has been found only in the whale-fall community at the “sulfophilic stage” (fueled by anaerobic breakdown of bone lipids) and has never been found elsewhere including shallow waters, wood-falls, cold seeps, or hydrothermal vents [7,15]. Therefore, it is natural to suppose that this lancelet may make use of the lipid-and-sulfide-rich or the organic material as a food source like other community members [26]. So far as the gross anatomy is concerned, there are no significant differences between *A. inferum *and its shallow-water congeners [7], which indicates that *A. inferum *may be a filter-feeder like its congeners. The mechanism of feeding of this whale-fall lancelet still remains as an open question.

When did *A. inferum *adapt to sulfide-rich environment? To examine the timing of *A. inferum *lineage, we estimated the divergence time between *A. inferum *and the other species of *Asymmetron *inhabiting coral reefs. With a slight difference between two resultant estimations of 115 Mya (Fig. 4) and 97 Mya (Fig. 5) (the middle Cretaceous), these appear far older than the first appearance of large whales, represented by the inshore archeocetacean *Basilosaulus *(40 Mya, the middle Eocene) [27]. Therefore, an *A. inferum*-like ancestor seems to have become a member of the whale-fall community after more than 57 million years of the emergence of *A. inferum *lineage (Figs. 4 and 5). Prior to the appearance of large whales, there were the Cenozoic or Mesozoic large fishes (e.g., the giant fossil shark *Carcharocles*, 10–20 m long; the ichthyodectid *Xiphactinus*, 4.2 m long) and the Mesozoic reptiles (e.g. ichthyosaurus [<16 m long] and plesiosaurus [<14 m long]) in the sea [4]. These large fishes and reptiles in the Mesozoic may have played an ecological role similar to the whales on the sea floor, as suggested by Martill et al. [28] and Hogler [29]. *A. inferum *may have been a member of these large vertebrate-fall communities before becoming a whale-fall specialist.

Another whale-fall specialist, the bone-eating marine worm *Osedax *also shows ancient divergence time (> 100 Mya) from its sister group (vestimentiferans + *Sclerolinum*) (inferred from [30]); however, the divergence time within *Osedax *(<42 Mya) [15,30,31] coincides with the first appearance of the large whales, based on the molecular clock. In the whale-fall bathymodiolin bivalves of the genera *Adipicola *and *Idas*, the divergence time (<30 Mya, the middle Oligocene) is synchronous with the major radiation of unequivocally oceanic whales [32,33], based on molecular age estimation (inferred from [26,34]) and the fossil records [35]. This synchronicity suggests that high sulfide-rich habitats derived from large whales with oil-rich bones [36] may play an important role in the divergences of *Osedax*, *Adipicola*, and *Idas*. Their tolerance and utilization of sulfide may have already been developed at the time of their invasion into whale-fall communities, because their sister groups are chemosymbiotic [26,30,34], provided actually or potentially with these abilities.

Unlike these whale-fall specialists, the *A. inferum*-like ancestor might have had a very low sulfide tolerance, because its sister group, *A. lucayanum *complex, and all the remaining lancelets inhabit aerobic environments [37]. This does not necessarily deny our hypothesis that *A. inferum *lineage may have started its life-mode as a vertebrate-fall specialist in the Mesozoic, because, unlike modern whale-falls with oil-rich bones, the Mesozoic large vertebrate-falls may possibly have lacked sulfophilic stages during decomposition [35,36], resulting in a low concentration of sulfide. Subsequently, the *A. inferum *lineage may have come to gain the sulfide tolerance to become a whale-fall specialist.

## Conclusion

The trees based on the whole mitogenome sequences show that the whale-fall specialist *A. inferum *is the sister group to the coral reef lancelet *A. lucayanum *complex, supporting the monophyly of the genus *Asymmetron*, assumed on the basis of the morphological characters. Furthermore, the features of the *A. inferum *mitogenome expand our knowledge of variation within cephalochordate mitogenomes, adding new cases of inversion and transposition of the *trnQ *gene. Novel gene orders in *Asymmetron *were found to be useful for phylogenetic inference.

The divergence time between *A. inferum *and its sister group *A. lucayanum *complex is estimated to be 115 Mya or 97 Mya (the middle Cretaceous), which is older than the first appearance of large whales (40 Mya, the middle Eocene). Our results raise the possibility that *A. inferum *lineage was initially a member of the Mesozoic and the early Cenozoic large vertebrate-fall communities, and subsequently shifting to become a whale-fall specialist.

## Methods

### Specimens and DNA extraction

Specimens of *A. inferum *were collected from the sediments underneath the bones of a sperm whale off Cape Nomamisaki, Kagoshima Prefecture, Japan, 229 m deep (Fig. 1) during the Hyper-Dolphin/Natsushima cruise of JAMSTEC (NT03-08 leg1). Comparative specimens of two species of *A. lucayanum *complex were collected with the aid of SCUBA from coral sand 10–20 m deep off the northwest coast of Kuroshima Island, Yaeyama Islands, Japan, and Castle Harbour in Bermuda [8]. These collected specimens were fixed and preserved in 99.5% ethanol. The whole body of a specimen from each species was used to extract total DNA, which was performed using the DNeasy Tissue Kit (QIAGEN), according to manufacturer's protocols.

### PCR and Sequencing

The mitogenomes of the three *Asymmetron *species were amplified in their entirety using a long PCR technique. Four lancelet-versatile long-PCR primers [see Additional File 1] were used to amplify the entire mitogenome in two reactions. The long-PCR products were diluted with TE buffer (1:19) for subsequent use as PCR templates. Forty-three lancelet-versatile, 39 fish-versatile, and 23 species-specific PCR primers [see Additional File 1] were used in various combinations to amplify contiguous, overlapping segments of the entire mitogenome. Fifty species-specific primers were designed in cases where no appropriate lancelet-versatile primers were available for *A. inferum*. Long PCR and subsequent nested PCR were performed as previously described [16]. Double-stranded PCR products, purified using a Pre-Sequencing Kit (USB), were subsequently used for direct cycle sequencing with dye-labeled terminators (Applied Biosystems). Primers used were the same as those for PCR. All sequencing reactions were performed according to the manufacturer's instructions. Labeled fragments were analyzed on a Model 3100 DNA sequencer (Applied Biosystems).

### Alignments

Four *Asymmetron*, an *Epigonichthys*, and three *Branchiostoma *species, including whole mitogenomes of known species (*Asymmetron *sp. B, AB110092[16]; *E. maldivensis*, AB110093[16]; *B. floridae*, AF098298[18]; *B. lanceolatum*, AB194383 [38]; *B. belcheri*, AB083384 [Matsuzaki et al., unpublished data]) were phylogenetically analyzed, based on surveyed mtDNA sequence data. An enteropneust *Balanoglossus carnosus *(AF051097[39]), a cyclostome *Petromyzon marinus *(U11880 [40]), and a small-spotted catshark *Scyliorhinus canicula *(X16067[41]) were chosen as outgroups. Urochordates were not included in the present analysis because of peculiarity of their mitogenome sequences that was remarkably different from those of other chordates, supposedly because of rapid evolutionary rate in the mitogenome [42,43].

The DNA sequences for the 11 species were edited and analyzed with EditView ver. 1.0.1, AutoAssembler ver. 2.1 (Applied Biosystems), and DNASIS ver. 3.2 (Hitachi Software Engineering Co. Ltd.). Amino acids were used for alignments of the protein-coding genes, and secondary structure models were used for the alignment of tRNA genes. Since strictly secondary-structure-based alignment for the two rRNA genes was impractical for the large dataset, we employed machine alignment instead, which would minimize erroneous assessment of the positional homology of the rRNA molecules. The two rRNA gene (*rrnL *and *rrnS*) sequences were initially aligned using CLUSTAL X, ver. 1.81 [44]. Each primary aligned sequence was realigned using ProAlign ver. 0.5 [45] and those regions with posterior probabilities ≥70% used in the phylogenetic analyses. These probabilities seemed to effectively remove all ambiguously aligned regions. Ambiguous alignment regions, such as the 5' and 3' ends of several protein-coding genes and loop regions of several tRNA genes, were excluded, leaving a total of 12,497 available nucleotide positions (10,059, 1,275, and 1,163 positions for protein-coding, tRNA, and rRNA genes, respectively) for phylogenetic analyses. Two datasets were used in our analyses: dataset #1, concatenated nucleotide sequences from 13 protein-coding, 22 tRNA, and two rRNA genes (total position 12,497); dataset #2, concatenated amino acid sequences from 13 protein-coding genes plus nucleotide sequences from 22 tRNA and two rRNA genes (5,791).

### Phylogenetic analysis

Maximum-likelihood (ML) analysis for dataset #1 using PAUP* 4.0b10 [46] was performed under a transversional substitution model with gamma correction and invariable-site assumption (TVM + *I *+ *Γ*), which was chosen as the most fit for the present case based on hierarchical likelihood tests by Modeltest 3.6 [47]. The base frequencies were estimated to be A = 0.2940, C = 0.2233, G = 0.1598, and T = 0.3230. The substitution rates were A-C = 0.9657, A-G = 8.4537, A-T = 1.3911, C-G = 1.6808, C-T = 8.4537, and G-T = 1.0000. Assumed proportion of invariable sites was 0.1312. Gamma distribution shape parameter was 0.4086. Heuristic search option of PAUP* was chosen for obtaining the ML tree. Robustness of each internal branch of the ML tree estimated was evaluated with 100 bootstrap replications [48].

Partitioned Bayesian inference (BI) phylogenetic analysis was performed with MrBayes version 3.1.2 [49,50]. Five (dataset #1) and three (dataset #2) partitions were set (1st, 2nd, 3rd codon positions, tRNA genes, and rRNA genes; and amino acid sequences of 13 protein-coding genes, tRNA genes, and rRNA genes, respectively). The general time reversible (GTR) model with gamma correction and invariable-site assumption was used in the analysis for dataset #1, and for tRNA and rRNA genes of dataset #2. As mentioned above, TVM + *I *+ *Γ *was chosen as the best fitted for the present case. However, the TVM model is a special case of the GTR model and is not yet implemented in MrBayes. Therefore, the GTR model (GTR + *I *+ *Γ*) was used in the analyses. The mtREV [20] model with gamma correction and invariable-site assumption (mtREV + *I *+ *Γ*) was used in the analysis for the protein-coding genes of dataset #2. This model was selected as the best-fit model of amino acid substitution by MrBayes. Model parameter values were treated as unknown and were estimated for each analysis. Random starting trees were used, and analyses were run for one million generations, sampling every 100 generations. Bayesian posterior probabilities were then calculated from the sample points after the Markov Chain Monte Carlo (MCMC) algorithm began to converge. To ensure that our analyses were not trapped in local optima, four independent MCMC runs were performed. Topologies and posterior clade probabilities from different runs were compared for congruence.

Maximum parsimony (MP) analysis for dataset #1 was performed using PAUP* 4.0b10 [46]. Heuristic MP analyses were conducted with TBR (tree bisection-reconnection) branch swapping and 100 random addition sequences. All phylogenetically uninformative sites were ignored. Robustness of each internal branch of the MP tree estimated was evaluated with 1,000 bootstrap replications [48].

### Divergence time estimation

The analyses of divergence time were conducted with the penalized likelihood (PL) [22] and the nonparametric rate smoothing (NPRS) [23] methods. Molecular clock approaches were not used because a high rate of heterogeneity among lineages of lancelets was observed by the two-cluster test (LINTREE [21]). The previous analyses based on molecules were referred to the calibration points for our dating because of the absence of a useful fossil record in the lancelets. PL approach based on the BI tree (dataset #2) was performed by r8s 1.71 [51]. All r8s analyses utilized the truncated Newton (TN) algorithm and the additive rate penalty function. All analyses were reoptimized 1,000 times (set_num_restarts = 1,000) to avoid entrapment on a local solution optimum. The optimal smoothing parameter (121) was estimated using cross-validation. The divergence times between Cephalochordata and Vertebrata (+ Urochordata) (891 Mya) and between Agnatha (Cyclostome) and Gnathostomes (Chondrichthys) (652 Mya) [1] were used for the age of two calibration points. NPRS approach based on the ML tree was performed by TreeEdit 1.0 [52]. As a reference point for dating, the divergence time between *Asymmetron *and the other genera (162 Mya) was used for the age of root node [16].

## Authors' contributions

TK, MNi, and TN conceived and designed the research; MNo, YF, and TN collected materials; TK and MNo performed experiments; TK, MNo, and YY analyzed data, TK, MNi, and TN wrote the paper. All authors read and approved the final manuscript.

## Supplementary Material

Additional File 1 List of primers. List of primers used in the PCR and sequencing for all species of the genus Asymmetron. Click here for file
