# Supplementary material for: Phylogenetic position of a whale-fall lancelet (Cephalochordata) inferred from whole mitochondrial genome sequences
Source: BMC Evol Biol. 2007 Jul 31;7:127. doi: 10.1186/1471-2148-7-127 (PMC2034537; doi:10.1186/1471-2148-7-127)
Supplement: Additional File 1 — List of primers. List of primers used in the PCR and sequencing for all species of the genus Asymmetron. [file 1471-2148-7-127-S1.pdf]

Additional File 1

The list of primers used in the PCR and sequencing for all species of the genus *Asymmetron*

L primers	Sequence (5' → 3')	Ref.	H primers	Sequence (5' → 3')	Ref.
Long PCR primers (A. inferum-specific primer)					
Asin-LA-*cox1*-L	GCGGGCAAGCATCGAATGAAATCGTGTTCC		Asif-LA-*rrnL*H	GGACAAGTGATTATGCTACCTTTGCACAGTC	
Long PCR primers (A. lucayanum-specific primer)					
			AlH6890-COI (981)	GGCGTTTCTCATTTAATCTGCTTAGATCCT	a
Long PCR primers (lancelet-versatile primers)					
AmphLAL1076-*cox1*	CTGTAGGTGGGTTAACAGGAATTGTATTAGC	a	AmphLAH916-*cox1*	CCTGTTGGTACAGCAATTACTATAGTAGCAGC	a
AmphLAL11947-*rrnL*	GACTGTGCAAAGGTAGCATAATCACTTGCCC	a	AmphLAH12317-*rrnL*	CTAGGAATCTTGATCCAACATCGAGGTCGC	a
Long PCR primers (fish-versatile primers)					
S-LA-16SL (12411)	CGATTAAAGTCCTACGTGATCTGAGTTCAG	b			
PCR and sequencing primers (A. inferum-specific primer)					
Asin-*cox1*-L1	TTCTTTGGTCACCCAGAGGT		Asin-*cox2*-H	TCGGTATATTCATAGCTTCAG	
Asin-*atp6*-L	ACGCAATGCTTCTCTCTTAG		Asin-*nad4L*-H1	GAGTGCTAAACCTGAACTGG	
Asin-*nad4*-L1	ACCTTTGAGGCCCTATAAAC		Asin-*nad4*-H	GTCTACTTGTAAGTCAAGCGG	
Asin-*nad4*-L2	ATTCTACACTTAACACCAGC		Asin-*trnH*-H	TGCCATTGGGATTAAATAAA	
Asin-*nad5*-L1	AGACAAATAGTAACACGTCAC		Asin-*nad6*-H	CGGGTAGAGCTGTTTTGTAA	
Asin-*nad5*-L2	TTTAGGGGTCATAAAAGCAA		Asin-*cob*-H	AATGTACAATAGCTAGTCCTG	
			Asin-*nad2*-H	TGCTCCGATTGATTGATTGAG	
PCR and sequencing primers (A. lucayanum-specific primer)					
Aslu(spA)-*nad5*-L	TGTTCATTTTGCAAGCCATG		AlH6012-COI (109)	GGCTGGGATAGTTCTGCWCGAAT	a
El-L6744-*nad6*	TTCGATCTCTATGAGCGCGG	a	El-H7103-*nad6*	GATTCTGCCTCTAATGTGGC	a
El-L6994-*nad6*	TAAAGACGTTTGCGCTTGAG	a	Aslu(spA)-*nad5*-H	AGAGATGCAAATACTGCGGC	
			El-H8317-*nad5*	ATACTAATTCTAGTCAGCGC	a
PCR and sequencing primers (lancelet-versatile primers)					
AmphL1325-*cox1*	GGNATACCWCGNCGRTATTCNGA	a	AmphH131-*cox1*	GGCTGNGANARTTCNGCNCGAAT	
AmphL1573-*trnS*	GTCTATTGGTTTCAAGCC	a	AmphH1580-*trnS*	TATGCGATTGGCTTGAAACC	a
AmphL2429-*trnK*	GCGTTAACCTTTTAAGTTAATGATGAG	a	AmphH2005-*cox2*	TCTGTRTAYTCRTARCTTCA	a
AmphL3328-*cox3*	CAYCCRTGRCAYTTAGTDGARCC	a	AmphH2403-*trnK*	CTCATCATTAACTTAAAAGGTTAACGC	a
AmphL4471-*trnR*	ATTTGGTTTCGGCCCANAAG	a	AmphH4049-*cox3*	CCTCACCAGTAAATACAWACG	a
AmphL5013-*nad4*	CCTTTGATATTAATTGCTAGHCAAC	a	AmphH4452-*trnR*	CTTNTGGGCCGAAACCAAAT	a
AmphLAL5212-*nad4*	GGAGCACAAAAAGAGCGTTATCAAGCAGGAACATA	a	AmphH4989-*nad4*	GTTGGCTAGCAATTAATATYAAAGG	a
AmphL5429-*nad4*	GGCTGCCTAAGGCTCAYGTDGAG	a	AmphH5407-*nad4*	CTCCACGTGTGCCTTWGGMARCC	a
AmphL6166-*trnH*	CTAGTTTAATWAGAATWCTAAGTTGTGGTC	a	AmphH6137-*trnH*	GACCACAACTTAGWATTCTWATTAAACTAG	a

AmphL6244-*trnS*	ATGTTGCTACCRTRRYYNATTARAGTTC	a	AmphH6217-*trnS*	GAACTTTAATNARTTATGGTAGCAACAT	a
AmphL7442-*nad5*	TGTTTCTTTATTGGKAGTGC	a	AmphH8842-*trnE*	ATAACRYAAGCTTTTCATGC	a
AmphL8307-*trnG*	AGAYTTCCAMTCTRARGRTCC	a	AmphH9081-*cob*	ACATCWCGRCARATATGNGC	a
AmphL8842-*trnE*	GCATGAAAARCTTRYGTTAT		AmphLAH9480-*cob*	CCTGTTGGATTATTKGCCCCTGTTTGRTGTAA	a
AmphLAL9412-*cob*	GGGYTTCTCYGTTGATAATGCMACACTAACTCG	a	AmphH10129-*trnP*	TAARCTTTGGGAGTTTAARAYG	
AmphL10108-*trnP*	CRTYTTAAACTCCCAAAGYTTA		AmphH11475-*rrnL*	CATTTCTTAARYAACCAGCTA	
AmphL10383-*rrnS*	GTGCCAGCCGCCGCGGTTA		AmphH12522-*trnL*	GCTTAGGCCTTWYGCAATAC	a
AmphL11103-*trnV*	TAAGCGTYTCTTTTACACGG		AmphH13633-*trnM*	CTCATGGTAGAGGATGGATT	a
AmphLAL11918-*rrnL*	GACTGTGCAAAGGTAGCATAATCACTTGCCC				
AmphL13396-*nad1*	TGRTTTCGKGCWTCNTACCC	a			
AmphL14219-*nad2*	GGAGGNYTNGGNCARACRCAANTRCG				
AmphL14488-*trnN*	CATCKTTGGATTAACAGTC	a			
AmphL14842-*trnW*	AGACCTTTAGCCTTCAAAGCTG				
PCR and sequencing primers (fish-versatile primers)					
L6631-COI (726)	TGRTTTTTGGTCACCCTGAAGT	a	H7227-COI (1324)	CATGTAGTGTATGCATCAGGGTARTC	g
L6730-COI (805)	TATATAGGAATRGTMTGAGC	b	H7892-COII (1996)	TCGTAGCTTCAGTATCATTG	a
L7255-COI (1352)	GATGCCTACACMCTGTGAAA	b	H8168-COII (2272)	CCGCAGATTTCWGAGCATTG	b
L7905-COII (2009)	GGCCAYCARTGGTAYTGAAG	a	H8589-ATP6 (2767)	AAGCTTAKTGTCATGGTCAGT	b
L8343-Lys (2402)	AGCGTTGGCCTTTTAAGCTAAWGATWGGTG	a	H9076-ATP6 (3144)	GGGCGGATAAAKAGGCTAAT	b
L8894-ATP6 (2879)	TTGGACTACTWCCSTATAC	b	H10244-ND3 (4275)	AGGAGSGCGATTTCWAGRTC	a
L9514-COIII (3599)	TTCTGAGCCTTCTAYCA	b	H10433-Arg (4458)	AACCATGGWTTTTTGAGCCGAAAT	b
L11895-ND4 (5899)	CCTAACCTWATGGGRGAACT	b	H11618-ND4 (5622)	TGGCTGACKGAKGAGTAGGC	c
L13280-ND5 (6749)	CAACTWGGKCTAATAATAGT	a	H12145-His (6138)	CTAGTGTTTTKGTTAAACTA	b
L15927-Thr (10062)	AGAGCGTCGGTCTTGTAAKCCG	c	H12632-ND5 (6586)	GATCAGGTTACGTAKAGKGC	b
L1085-12S (10530)	TAAACCAGGATTAGATACCC	d	H14086-ND5 (7764)	AGGSTKAGGTAKGTTTTRAT	c
L1496i-12S (10909)	GTACATATCGCCCGTCGCTT	e	H1358-12S (10691)	CGACGGCGGTATATAGGC	b
L2188-16S (11601)	AGTGGGCCTAAAAGCAGCCA	a	H1478-12S (10842)	GAGAGTGACGGGCGATGTGT	d
L2510i-16S (11855)	CGCCTGTTTAACAAAAACAT	f	H1903-16S (11341)	GTAGCTCGTYTAGTTTCGGG	b
S-LA-16SL (12411)	GATTAAAGTCCTACGTGATCTGAGTTCAG	b	H2491c-16S (11836)	ATGTCTTTGTTAAACAGGCG	a
L3686-ND1 (12964)	TGAGCMTCWAATTCMAAATA	a	H2716i-16S (12051)	AAGTTTTATAGGGTCTTATCGTC	e
L4180-ND1 (13437)	CAACTCATGCATTTRGTWTGRAAAA	c	H3466-ND1 (12744)	ATKGGTTCTTTGATGAAKAGTTT	c
L4438-Met (13618)	AAGCTTTTGGCCCCATRCCC	a	H3976-ND1 (13254)	ATGTTGGCGTATTCKGCKAGGAA	a
			H5622-Ala (14916)	TTAATTAAAGTGTCTGKKTTGCA	a
			H4557-ND2 (14752)	GTATTAATTTCTAGKCCTATTCA	a

a, Nohara et al. (2005); b. Miya and Nishida (1999); c, Miya and Nishida (2000); d, Miya and Nishida (1998); e, Kitaura, Wada and Nishida (1998); f, Palumbi et al. (1991); g, Nishida, Ohkawa and Iwata (1998)

References

Kitaura J, Wada K, Nishida M: **Molecular phylogeny and evolution of unique mud-using territorial behavior in ocypodid crabs (Crustacea: Brachyura: Ocypodidae)**. *Mol Biol Evol* 1998, **15**(6):626-637.

Miya M, Nishida M: **Molecular phylogeny and evolution of the deep-sea fish genus *Sternoptyx***. *Mol Phylogenet Evol* 1998, **10**(1):11-22.

Miya M, Nishida M: **Organization of the mitochondrial genome of a deep-sea fish, *Gonostoma gracile* (Teleostei: Stomiiformes): First example of transfer RNA gene rearrangements in bony fishes**. *Mar Biotechnol* 1999, **1**(5):416-426.

Miya M, Nishida M: **Use of mitogenomic information in teleostean molecular phylogenetics: A tree-based exploration under the maximum-parsimony optimality criterion**. *Mol Phylogenet Evol* 2000, **17**(3):437-455.

Nishida M, Ohkawa T, Iwata Y: **Methods of analysis of genetic population structure with mitochondrial DNA markers**. *Fish Genet Breed Sci* 1998, **26**:81–100. (in Japanese)

Nohara M, Nishida M, Miya M, Nishikawa T: **Evolution of the mitochondrial genome in Cephalochordata as inferred from complete nucleotide sequences from two *Epigonichthys* species**. *J Mol Evol* 2005, **60**:526–537.

Palumbi SR, Martin A, Romano S, McMillan WO, Stice L, Grabowski G: **The simple fool's guide to PCR. ver. 2**. Honolulu: University of Hawaii Press; 1991.
